# Diagnosis of Familial Hypercholesterolemia in Children and Young Adults

**DOI:** 10.3390/ijms25010314

**Published:** 2023-12-25

**Authors:** Olga Timoshchenko, Dinara Ivanoshchuk, Sergey Semaev, Pavel Orlov, Valentina Zorina, Elena Shakhtshneider

**Affiliations:** 1 Institute of Internal and Preventive Medicine (IIPM)–Branch of ICG SB RAS, 175/1 Borisa Bogatkova Str., Novosibirsk 630089, Russiashakhtshneyderev@bionet.nsc.ru (E.S.); 2 Institute of Cytology and Genetics, Siberian Branch of Russian Academy of Sciences (ICG SB RAS), 10 Prospekt Ak. Lavrentyeva, Novosibirsk 630090, Russia

**Keywords:** familial hypercholesterolemia, children, young adults, molecular genetic testing

## Abstract

The early detection and treatment of familial hypercholesterolemia (FH) in childhood and adolescence are critical for increasing life expectancy. The purpose of our study was to investigate blood lipid parameters, features of physical signs of cholesterol accumulation, and a personal and family history of premature cardiovascular diseases in children and young adults when FH is diagnosed. The analysis included patients under 18 years of age (n = 17) and young adults (18–44 years of age; n = 43) who received a diagnosis of FH according to clinical criteria. Targeted high-throughput sequencing was performed using a custom panel of 43 genes. A family history of cardiovascular diseases was more often noted in the group under 18 years of age than in young adults (*p* < 0.001). Among young adults, there was a high prevalence of typical signs of the disease such as tendon xanthomas and the early development of arterial atherosclerosis (*p* < 0.001). By molecular genetic testing, “pathogenic” and “probably pathogenic” variants were identified in the genes of 73.3% of patients under 18 years of age and 51.4% of patients 18–44 years of age. Thus, blood lipid screening tests combined with an accurate assessment of the family history is a highly relevant and inexpensive option for diagnosing FH in childhood. Molecular genetic testing allows us to make an accurate diagnosis and to improve adherence to treatment.

## 1. Introduction

Familial hypercholesterolemia (FH) is a common lipid metabolism disorder caused by genetic defects that reduce the rate of the removal of low-density lipoprotein cholesterol (LDL-C) from the bloodstream and significantly increase the total cholesterol (TC) concentration in the blood. In patients with FH, starting from birth, elevated concentrations of TC and LDL-C in blood serum are observed, with normal or moderately increased triglyceride (TG) levels [1,2]. There are heterozygous (heFH) and homozygous FH (hoFH). The level of TC in patients with heFH, when there is a genetic defect inherited from one of the parents, usually is 7.5–14.0 mmol/L. In hoFH, in which the genetic defect is inherited from both parents, this parameter is 14–26 mmol/L [1].

According to a meta-analysis from 2017, which included data from 19 studies and 2.5 million people, the prevalence of heFH in the world is 0.40% (95% confidence interval: 0.29% to 0.52%), which corresponds to 1 in 250 people [3]. According to a larger meta-analysis by S.O. Beheshti et al. (2020), who combined data from 11 million people, the prevalence of FH is 1 case per 313 people. FH prevalence was also found to be 10 times higher among patients with coronary heart disease (CHD), 20 times higher among patients with premature CHD, and 23 times higher among patients with severe hypercholesterolemia [4]. The prevalence of the disease seems to vary among ethnicities and geographic areas, with a higher prevalence reported in subpopulations with founder effects or communities sharing ascendants or in those with higher rates of consanguinity (e.g., Afrikaners in South Africa, the Christian Lebanese, Tunisians, and some French Canadians) [5]. For example, among French Americans, the prevalence of FH in the province of Quebec is twice that of most population samples. At the time of the 1981 census, regional prevalence estimates were 1:154 for northeastern Quebec and 1:80 on the north bank of the St. Lawrence River. Based on initial results from the Canadian FH Registry and a recent meta-analysis of the prevalence of FH, it has been estimated that there are approximately 145,000 people with FH in Canada. This phenomenon is due to the founder effect, characterized by a decrease in genetic diversity because a relatively small number of individuals with genetic mutations causing FH create autonomous populations [6]. FH prevalence is unknown in 90% of the world’s countries [4]. In the study “Epidemiology of cardiovascular risk factors and diseases in regions of the Russian Federation”, it was shown in various parts of Russia that in two regions of Siberia (Tyumen and Kemerovo Oblasts), the prevalence of FH is 1 per 108 people, whereas in Primorsky Krai, it is 1 per 172 people [7]. The prevalence of the disease in Siberia is not explained by the founder effect and is mainly due to dietary patterns, which aggravate the clinical manifestation of the disease. The prevalence of FoHS is much lower, varying among countries from 1 in 300 thousand to 1 in a million people [1]. In Europe, there are up to 4.5 million people with FH, of whom 20–25% are children and adolescents. Individuals with FH are at an extremely high risk of coronary or other cardiovascular diseases (CVDs), and if FH is left untreated, then such diseases may develop in childhood or adolescence [8]. The early detection and treatment of FH in childhood/adolescence are critical for extending life expectancy. Members of the Global Familial Hypercholesterolemia Community and coauthors report that FH is currently largely underdiagnosed and undertreated, with only 10% of FH cases having been diagnosed and adequately treated [9].

The main cause of the disease is the presence of a pathogenic variant in the *LDLR* gene in almost 90% of patients with genetically confirmed FH. In approximately 10% of patients with FH, the cause is a pathogenic variant in the gene encoding apolipoprotein B (*APOB*): the main protein of LDL particles. In less than 1% of FH cases, pathogenic variants are detected in the gene encoding proprotein convertase subtilisin/kexin type 9 (PCSK9), which participates in LDL-C upregulation in the blood, and clinically important mutations in this gene enhance its function. In the rare autosomal recessive type of FH, homozygous pathogenic variants in the low-density lipoprotein receptor adapter protein 1 (*LDLRAP1*) gene can also lead to the phenotype of FH [1].

For the diagnosis of FH in childhood and adolescence, in accordance with Russian clinical guidelines, the Simon Broome criteria are recommended; for the diagnosis of FH in adults 18 years of age and older, the Dutch criteria are advisable (Dutch Lipid Clinic Network [DLCN] Criteria) [1]. Molecular genetic testing is recommended to confirm an FH diagnosis in individuals with a score of ≥6 on the DLCN clinical criteria or with a “definite” diagnosis according to the Simon Broome criteria [1]. FH often remains undiagnosed and untreated in children because the quantification of blood cholesterol and its fractions is not included in mandatory pediatric screening [10]. Difficulties in making this diagnosis are also associated with the fact that in children and adolescents with suspected FH, aside from an increased level of LDL-C, it is often not possible to identify other phenotypic manifestations of hypercholesterolemia: xanthomas, a corneal arcus, and atherosclerotic changes in arteries, especially in a case of heFH; furthermore, a family history of CVDs is not known in every case [11]. Therefore, cascade screening for FH in children is relevant as the most effective way to identify new patients with FH [1,12]. In the United States, selective screening is recommended starting at age 2, and universal screening starting from 9–11 years of age. Universal screening means the screening of all persons under 20 years of age [13].

The purpose of our work was to investigate blood lipid parameters, features of physical signs of cholesterol accumulation, and a personal and family history of premature CVDs in children and young adults when FH is diagnosed.

## 2. Results

### 2.1. The Main Characteristics of the Study Sample

In the group of patients under 18 years of age, the diagnosis of FH was made based on the Simon Broome clinical criteria: 71% of the group was diagnosed with “definite FH” and 29% as “probable FH”. In the group of patients over 18 years of age, the median [interquartile range] score according to the DLCN criteria was 6 [4; 9]. The diagnosis of definite FH (>8 points) was made in 23% of these patients, probable FH (6–8 points) in 42% of the patients, and “possible FH” (3–5 points) in 35%.

Patients under 18 years of age were more likely to have a family history of CVDs than young adults (94% of the group versus 88%, *p* < 0.001) (Table 1). During the medical examination, tendon xanthomas were two times more common among middle-aged people than among patients under 18 years of age (23% versus 12%, *p* < 0.001); none of the examined patients had a corneal arcus. In 28% of patients aged 18–44 years, according to previous ultrasonographic analyses, atherosclerosis of brachiocephalic arteries was confirmed. None of the children had a history of CHD or arterial atherosclerosis.

The maximal level of TC before the start of lipid-correcting therapy—as reported by the patients—did not differ between the age groups: 8.05 [6.43; 9.98] and 8.24 [6.8; 10.2] mmol/L (*p* = 0.525). When the lipid profile was evaluated, it was determined that the two groups were comparable in levels of TC, LDL-C, HDL-C, and lipoprotein A. TG levels were higher in young adults than in children (1.08 [0.82; 1.93] vs. 0.68 [0.55; 0.91] mmol/L, *p* = 0.005).

Thus, for persons under 18 years of age, it is more common to have a history of CVD and the absence of phenotypic signs of hypercholesterolemia and arterial atherosclerosis. Young people over 18 years are characterized by the presence of tendon xanthomas and arterial atherosclerosis.

### 2.2. Results of Molecular Genetic Analyses of Children with FH

In this work, a molecular genetic analysis was performed on 15 out of 17 patients under 18 years of age. “Pathogenic” and “likely pathogenic” variants were identified in 73.3% (n = 11) of the patients under 18 years of age (Table 2).

In all patients, the identified pathogenic and likely pathogenic variants were in a heterozygous state.

### 2.3. A Clinical Case

**Figure 1:**
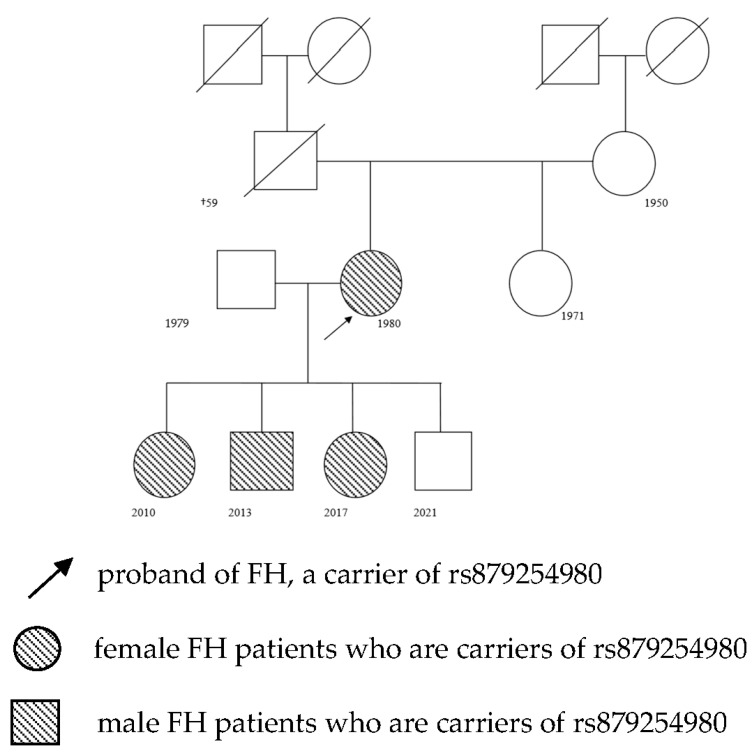
The screened family with variant rs879254980 identified in the *LDLR* gene.

A female proband (subject P5), 40 years old, visited the Lipid Center of the IIPM (a branch of the ICG SB RAS) with complaints of an increase in TC concentration starting from the age of 35 years up to maximal values of TC of 18 mmol/L and LDL-C of 15 mmol/L (Figure 1). The patient voluntarily signed an informed consent form for the publication of personal medical information in an anonymized form. Before visiting the Lipid Center, statin therapy (atorvastatin and rosuvastatin) was carried out in various doses, with an insufficient therapeutic effect. The family history contains CVDs in the form of ischemic stroke at ages 55 and 56 years in the father and his death from myocardial infarction at age 59. During the medical examination of the proband, the thickening of the Achilles tendons by up to 20 mm on both sides was revealed. Ultrasonography of brachiocephalic arteries and arteries of the lower extremities detected no atherosclerotic plaques, and the intima–media complex was found to not be thickened. Biochemical blood tests before the start of lipid-lowering therapy yielded the following results: TC: 16.1 mmol/L, LDL-C: 14.3 mmol/L, TGs: 1.2 mmol/L, HDL-C: 1.3 mmol/L, alanine aminotransferase (ALT): 16 U/L, glucose: 5.3 mmol/L, and creatine phosphokinase: 132 U/L. According to the clinical criteria of the DLCN (score of 15), a diagnosis of definite FH was made.

As part of cascade screening, the mother and children of the proband were examined too.

A daughter of the proband, subject P6, was born in 2010. An examination revealed no signs of FH. Biochemical blood tests yielded the following results: TC: 12.1 mmol/L, LDL-C: 10.3 mmol/L, TGs: 0.6 mmol/L, and HDL-C: 1.7 mmol/L.

A son of the proband, subject P4, was born in 2013. An examination revealed a thickening of the Achilles tendons on both sides by up to 1.5 mm. Biochemical blood tests gave the following results: TC: 9.7 mmol/L, LDL-C: 7.9 mmol/L, TGs: 0.7 mmol/L, and HDL-C: 1.3 mmol/L.

Another daughter of the proband, subject P7, was born in 2017. An examination revealed a thickening of the right Achilles tendon by up to 1.3 mm. Biochemical blood test results were as follows: TC: 10.7 mmol/L, LDL-C: 9.1 mmol/L, TGs: 0.7 mmol/L, and HDL-C: 1.2 mmol/L.

The son born in 2021 was not examined.

Molecular genetic testing was performed, revealing pathogenic variant rs879254980 in the *LDLR* gene in the proband (subject P5) and in all examined children (subjects P4, P6, and P7) in a heterozygous state (Table 2). In the proband’s mother, who did not have clinical manifestations of FH and had normal blood lipid levels, this pathogenic variant was not found.

On the basis of the medical history, medical examination data, laboratory test results, and molecular genetic findings, the proband received a diagnosis of dyslipidemia IIA (by Fredrickson) and definite FH in a heterozygous state (DLCN criterion 15b). Cardiovascular risk was high. The target level of LDL-C was <1.8 mmol/L.

The proband was given recommendations on following a lipid-lowering diet and was prescribed a combination lipid-lowering therapy: rosuvastatin at 40 mg/day and ezetimibe at 10 mg/day. Considering the failure to attain the target level of LDL-C (7.4 mmol/L), according to recommendations from Russian clinical guidelines for the diagnosis and treatment of lipid metabolism disorders [14], evalocumab at 140 mg once every 2 weeks was added to the treatment regimen. During the triple lipid-lowering therapy in the proband, a reduction in LDL-C by 70% of initial values (4.3 mmol/L) was achieved successfully, but the target level was not reached.

In cooperation with a pediatrician and nutritionist, individual diets were selected for the proband’s children. According to national clinical guidelines for the management of patients with FH [1], the patient’s two children (the daughter born in 2010 and the son born in 2013) were advised to take atorvastatin at a dose of 10 mg/day. The parents, however, decided to refrain from starting statins until the children reached adulthood, and hence the patients were prescribed ezetimibe at 10 mg/day.

### 2.4. Results of the Molecular Genetic Testing of Young Adults with FH

Of the 42 patients 18–44 years of age, molecular genetic research was performed on 37 patients. Pathogenic and likely pathogenic variants were found in 51.4% (n = 19) of these patients (Table 3).

Among patients with a score of ≥6 on the DLCN criteria, the prevalence of pathogenic variants was 84.2% (n = 16), whereas among patients with the score < 6, the prevalence of pathogenic variants was 15.8% (n = 3).

### 2.5. Treatment of Patients under the Age of 18 with FH

In our study, for all patients under the age of 18 years, with the help of a physician–nutritionist, an individualized low-fat diet was selected. Beginning at the age of 10 years (n = 10), a lipid-lowering therapy was prescribed: atorvastatin at an initial dose of 10 mg with the monitoring of levels of hepatic transaminases, creatine phosphokinase, and glucose. At the follow-up visit 1 month later, only two (20%) children were taking statins. The parents of the other children, despite good awareness of the disease and professed adherence to and interest in treatment, decided to postpone statin therapy until the children reached adulthood. According to clinical guidelines [1,14], they were recommended ezetimibe at 10 mg/day.

All children with FH are kept under observation by cardiologists. Explanatory conversations are regularly held with parents about the need to take lipid-lowering medication. For patients, there are accessible Internet resources for people with hereditary dyslipidemia, including children. In our study, all patients in the age group of 18–44 years received lipid-lowering therapy while following a diet. Combination therapy with a statin at a tolerated dose and ezetimibe was received by 35% of the patients; the remaining patients took atorvastatin or rosuvastatin at a maximum tolerated dose. As many as 72% of the patients achieved target LDL cholesterol levels during the treatment.

## 3. Discussion

According to the results of our study, patients under 18 years of age were more likely to have a family history of CVD than in the group of young people 18–44 years of age. These differences are probably due to the fact that all children and adolescents were examined as part of cascade screening with a known hereditary history of CVDs in their parents, in contrast to the older patients, who applied to our clinic, including on their own. To diagnose FH in children, it is extremely important to focus on collecting a family history of TC and LDL-C levels and of early CVDs not only in parents, but also in second-degree relatives [15].

A comparison of the FH phenotype between young adults and children revealed a higher prevalence—in young adults—of typical features such as tendon xanthomas and the early development of arterial atherosclerosis (*p* < 0.001). This difference may be explained by time-limited exposure to high levels of LDL-C (lower TC load), resulting in the absence of typical signs of FH in children. In another study, after an analysis of 170 children (age 5.71 ± 0.71 years, mean ± SD) with a genetically confirmed diagnosis of FH, scientists from Slovenia Urh Groselj et al. did not find tendon xanthoma, xanthelasma, a corneal arcus, or CHD in any patient [16]. It has also been reported that in a Portuguese cohort of 237 children with FH (age 10.0 ± 3.6 years; range 2–17 years), none have CHD or tendon xanthoma [17].

The age groups under study here did not differ in terms of TC, LDL-C, HDL-C, and lipoprotein A, but TG levels were higher in young adults than in children (*p* = 0.005). Given that patients with secondary lipid disorders were excluded from our study, the higher levels of TGs in adult patients may be attributed to dietary patterns, including the consumption of alcoholic beverages and foods high in animal fat, but a detailed dietary assessment was beyond the scope of the study protocol. The diagnosis of FH in persons during puberty should take into account sex- and age-specific features of fluctuations of blood lipid concentrations. Throughout puberty, the levels of TC and LDL-C diminish, the concentration of TGs in males may rise, but the levels of HDL-C and TGs in females do not change [18]. Consequently, a diagnostic approach based primarily on LDL-C may result in the detection of only the most severe cases with very high lipid levels. Therefore, assessing the family history at a young age is becoming the most relevant approach.

Thus, because in children and adolescents the symptoms of hypercholesterolemia are extremely mild, focusing the attention of practitioners on a thorough collection of family history will help to identify patients with FH and to promptly prescribe treatment.

In our work, pathogenic and likely pathogenic variants were identified in the genes of 73.3% (n = 11) of the patients under 18 years of age. Pathogenic and likely pathogenic variants were identified in 51.4% (n = 19) of the young adult patients. The vast majority of pathogenic variants were found in the *LDLR* gene in both groups. One patient proved to be a compound heterozygote for the *LDLR* gene. Rare, likely pathogenic variants in genes *APOB* and *ABCG5* in a heterozygous state were identified too.

In our previous study, the frequency of detected pathogenic and likely pathogenic variants was 47.5% among the examined adult probands (age 46 ± 13.9 years, range: 20–73 years) with an FH phenotype from Western Siberia (Russia) and 85.7% among the children of the probands [19]. As in other articles, this finding confirms the effectiveness of the cascade genetic screening strategy for diagnosing FH in the children and relatives of patients with FH.

This paper presents a clinical case of diagnosing FH by cascade genetic screening. After diagnosing FH, the proband’s family members were examined. Three out of four children of the proband received a diagnosis of lipid metabolism disorders for the first time. The FH diagnosis in the children was confirmed by molecular genetic testing.

The treatment and observation of children and adolescents with FH should be performed jointly by a pediatrician and a cardiologist [1]. The first step in the treatment of children with dyslipidemia is the choice of a diet. Therapeutic nutrition should ensure adequate caloric intake in accordance with sex- and age-specific energy needs, so as not to affect growth and neurohumoral development, while avoiding excessive restrictions and taking into account taste preferences [20]. As demonstrated by studies DISC and STRIP, a reasonable low-fat diet in childhood is effective and can help reduce plasma levels of TC and LDL-C by 10–15% of baseline values, while being safe for growth [21,22]. Lipids represent a concentrated source of energy and can be limited only partially: according to major studies in this field, daily lipid intake should not be lower than 25–30% of total energy [23]. Protein intake should provide a solid energy basis for every child’s growth, but an excess should be avoided. During development, the optimal protein intake should be 12–14% of the total daily amount of energy, with a 1:1 ratio of animal to plant proteins. Carbohydrates are the main source of energy and should account for 55–60% of total daily calories, while simple sugars should not exceed 10% [23]. It is extremely important that pediatric patients have a nutritious diet, that all macronutrients (protein, fat, and carbohydrates) are present in every meal, and that patients have an adequate micronutrient intake. The distribution of calories throughout the day is fundamental to maintaining harmonious growth without a deficiency or excess. The daily diet should be divided into four main meals. It is important to keep in mind that dietary recommendations for children with FH do not involve a strict and mandatory diet; they are based on nutritional recommendations that all children and adolescents should follow to lead a healthy lifestyle. The goal of the dietary intervention is to promote healthy lipid-controlled diets, including for members of the entire family, and thus to increase the chances of continued adherence to the diet into adulthood [24,25].

According to Russian recommendations for the treatment of patients with FH, the second stage in the treatment of children with FH is the prescription of a lipid-lowering pharmacotherapy, whose start is recommended at 8–10 years of age [1]. Pharmacotherapy should begin with the prescription of statins at low doses, with a gradual titration to optimal doses. In children with heFH, atorvastatin is allowed from 10 years of age, simvastatin is allowed from 10 years, and fluvastatin from 9 years. If target levels of LDL-C are not achieved when a maximum recommended or maximum tolerated dose of statins is taken, it is advisable to add 10 mg/day of ezetimibe. The target level of LDL-C in children 8–10 years old is <4.0 mmol/L, and in children over 10 years, it is <3.5 mmol/L. In the case of hoFH, aside from the above treatment, it is possible to prescribe inhibitors of PCSK9 (evalocumab in children over 12 years of age, subcutaneously 140 mg every 2 weeks or 420 mg once a month), whereas in especially severe cases, extracorporeal treatments can be prescribed (immunosorption of low-density lipoproteins [LDL apheresis] or cascade plasma filtration) [1,14]. Treatment with evalocumab in the United States is approved for patients with hoFH from 10 years of age. Severe FH, particularly hoFH, remains difficult to treat [26].

For all our patients under 18 years old, an individualized low-fat diet was selected. Starting at the age of 10 years (n = 10), statins were prescribed; however, at a follow-up visit a month later, only two (20%) children were in compliance with the treatment recommendations. Gisle Langslet et al. have conducted a retrospective study assessing adherence to lipid-lowering therapy among children and young adults with FH (n = 371). Low commitment was found in 30% of those surveyed, and the main reason was a lack of motivation. Older age, a greater number of visits, and the length of the follow-up were associated with good adherence [27].

Limitations of our study: the most common limitation of such studies is the small sample size.

Diagnostic problems are not the only critical aspect of the observation of children and adolescents with FH. Because of their age, patients under 18 years require a more purposeful specialized approach tailored to both the patient and the family, whether it be a dietary and lifestyle intervention or a pharmacotherapy. Molecular genetic testing is important and relevant both for diagnosis and for increasing adherence to treatment.

## 4. Materials and Methods

### 4.1. The Study Sample

Among the patients who visited the clinic at the Institute of Internal and Preventive Medicine (IIPM, a branch of the Institute of Cytology and Genetics [ICG], the Siberian Branch of the Russian Academy of Sciences [SB RAS]) for a consultation with a cardiologist–lipidologist while having lipid metabolism disorders, or among the patients called for an appointment as part of a cascade screening for FH, we enrolled patients under 18 years of age (n = 17, age 10 [7.5; 14] years: median [interquartile range]) and young adults aged 18–44 years (n = 43, age: 35 [28; 39] years) with an established diagnosis of FH according to clinical criteria. Patients with secondary dyslipidemias were not included in the study.

All patients had a medical history taken, a medical examination was performed, and the results of biochemical blood tests were obtained. When a personal anamnesis was collected, attention was focused on the data regarding the early development of a CVD and atherosclerotic lesions in cerebral/peripheral arteries (in males < 55 years old; in females <60 years old). The collected family history data included a history of myocardial infarction in a second-degree relative under the age of 50 years or in a first-degree relative under the age of 60, the presence of tendon xanthomas in a close relative, and the lipid profiles of relatives. In addition to the standard examination, the identification of possible tendon xanthomas and of a corneal arcus was carried out. Blood tests were performed on an empty stomach to evaluate the lipid profile (TC, LDL-C, high-density lipoprotein cholesterol [HDL-C], and TGs) and lipoprotein A, and a blood sample was taken for molecular genetic testing.

### 4.2. Molecular Genetic Analysis

To isolate DNA from blood, the phenol–chloroform extraction method was used [28]. The quality of extracted DNA was assessed by means of an Agilent 2100 Bioanalyzer capillary electrophoresis system (Agilent Technologies Inc., Santa Clara, CA, USA).

Targeted high-throughput sequencing (next-generation sequencing) was performed using a custom gene panel. The custom panel consisted of 43 genes (CDS + 100 bp padding) associated with FH and lipid metabolism disorders: *LDLR*, *APOB*, *PCSK9*, *LDLRAP1*, *CETP*, *LPL*, *HMGCR*, *NPC1L1*, *PPARA*, *MTTP*, *LMF1*, *SAR1B*, *ABCA1*, *ABCG5*, *ABCG8*, *CYP7A1*, *STAP1*, *LIPA*, *PNPLA5*, *APOA1*, *APOA5*, *APOC2*, *APOE*, *LCAT*, *ANGPTL3*, *LIPC*, *APOA4*, *APOC3*, *SREBF1*, *LMNA*, *PPARG*, *PLIN1*, *POLD1*, *LPA*, *SMAD1*, *SMAD2*, *SMAD3*, *SMAD4*, *SMAD5*, *SMAD6*, *SMAD7*, *SMAD9*, and *LIPG*. The targeted high-throughput sequencing was conducted using NimbleGen SeqCap Target Enrichment (Roche, Basel, Switzerland) on a MySeq sequencer (Illumina, San Diego, CA, USA). The coverage was 97%. Sequencing data analysis involved mapping the data to human genome assembly GRCh38. The automated processing and annotation of the obtained next-generation sequencing data was carried out on the NGS Wizard platform (genomenal.com, accessed on 22 May 2023). Rare “likely pathogenic” and “pathogenic” variants were verified by automated direct sequencing.

Data on clinical significance and on predictions of the pathogenicity of annotated single-nucleotide variants were retrieved from databases ClinVar and VarSome, in addition to the literature. Allele frequencies were annotated by means of databases GnomAD v3.1.2 [29] and RUSeq (http://ruseq.ru, accessed on 12 August 2023). The variants described in ClinVar or VarSome or predicted in silico to be benign/likely benign were excluded from the analysis. We assessed the pathogenicity of new variants according to the guidelines of the American College of Medical Genetics and Genomics (ACMG) and the Association for Molecular Pathology.

For patients without functionally significant substitutions in the panel genes, multiplex ligation-dependent probe amplification (MLPA) analysis was performed to determine structural changes (deletions or duplications) in the promoter and exons of the LDLR gene. The MLPA analysis was performed with SALSA MLPA Kit P062 (MRCHolland, Amsterdam, the Netherlands). For interpreting the results of MLPA, the Coffalyser.Net software (MRCHolland, Amsterdam, the Netherlands; https://www.mrcholland.com/technology/software/coffalyser-net, accessed on 24 May 2023) was employed.

### 4.3. Statistical Analyses

Statistical processing of data was conducted using the SPSS 19.0 software package. The type of a distribution of quantitative variables was determined by the Kolmogorov–Smirnov method. Because of the non-normal distribution of the characteristics under study, a median (Me) and 25th and 75th percentiles were calculated, and the quantitative variables in two groups were compared by the nonparametric Mann–Whitney *U* test. The χ^2^ test was performed to evaluate qualitative parameters. Differences were considered significant at *p* < 0.05.

## 5. Conclusions

The detection of FH in childhood is complicated by less severe physical manifestations of FH in the first decade of life, as a consequence of only short-term exposure to high blood concentrations of LDL-C. A screening assay of blood lipids in combination with an accurate assessment of a family history of CVDs is a very relevant and inexpensive option for diagnosing FH in childhood. Molecular genetic testing enables clinicians to make an accurate diagnosis and to improve adherence to treatment.

## Figures and Tables

**Table 1:** Characteristics of signs of FH in patients under 18 years of age and young adults.

Parameter	Age Groups		*p*
	<18 Years	18–44 Years	
Number of subjects	17	43	-
Men, n (%)	5 (29)	15 (35)	0.047
Age, years, Me [25th; 75th]	10 [7.5; 14]	35 [28; 39]	<0.001
Family history of CVDs, n (%)	16 (94)	38 (88)	<0.001
Body-mass index, kg/m^2^	19.3 [16.4; 23.2]	23.7 [21.6; 28.0]	0.008
Tendon xanthomas, n (%)	2 (12)	10 (23)	<0.001
Arterial atherosclerosis, n (%)	-	12 (28)	<0.001
Maximum level of TC the patient, mmol/L, Me [25th; 75th]	8.6 [6.6; 10.69]	9.4 [8.0; 10.96]	0.199
TC, mmol/L, Me [25th; 75th]	8.05 [6.43; 9.98]	8.24 [6.8; 10.2]	0.525
LDL-C, mmol/L, Me [25th; 75th]	5.85 [4.15; 7.55]	5.73 [4.84; 7.31]	0.717
HDL-C, mmol/L, Me [25th; 75th]	1.44 [1.17; 1.66]	1.39 [1.07; 1.91]	0.925
TG, mmol/L, Me [25th; 75th]	0.68 [0.55; 0.91]	1.08 [0.82; 1.93]	0.005
Lipoprotein a, mg/dL, Me [25th; 75th]	16.8 [14.1; 31.2]	23.7 [20.3; 65.1]	0.245

**Table 2:** Variants in FH-associated genes of patients under 18 years of age.

Patient ID	Position Number in the Reference Sequence	Gene	Position on Chromosome (GRCh38)	Nucleotide Substitution	Amino Acid Replacement	Minor Allele Frequency (MAF) According to the GnomAD Database	Clinical Significance According to the ClinVar Database
P82	rs121908038	*LDLR*	19:11113293	c.1202T>A	p.Leu401His	ND	Likely pathogenic
P60	rs879254566	*LDLR*	19:11105440	c.534TT>G	p.Asp178Glu	ND	Pathogenic/likely pathogenic
P55	rs879254721	*LDLR*	19:11107496	c.922G>A	p.Glu308Lys	ND	Pathogenic
P4, P6, P7	rs879254980	*LDLR*	19:11116179	c.1672G>T	p.Glu558Ter	ND	Pathogenic
P36, P37	rs879255191	*LDLR*	19:11128090	c.2389+5G>A	-	ND	Conflicting interpretations of pathogenic/likely pathogenic
P73	rs570942190	*LDLR*	19:11113337	c.1246C>T	p.Arg416Trp	T = 0.000007	Not reported in ClinVar
P22	rs5742904	*APOB*	2:21006288	c.10580G>A	p.Arg3527Gln	T = 0.000275	Pathogenic
P95	rs145164937	*ABCG5*	2:43832056	c.293C>G	p.Ala98Gly	C = 0.002223	Conflicting interpretations of pathogenicity/likely pathogenic

**Table 3:** Variants in FH-associated genes of patients 18–44 years of age.

Subject ID	Position No. in Reference Sequence	Gene	Position on Chromosome (GRCh38)	Nucleotide Substitution	Amino Acid Substitution	Minor Allele Frequency (MAF) According to GnomAD	Clinical Significance According to ClinVar
P5	rs879254980	*LDLR*	19:11116179	c.1672G>T	p.Glu558Ter	ND	Pathogenic
P34, P53	rs28942078	*LDLR*	19:11113376	c.1285G>A	p.Val429Met	A = 0.000012	Pathogenic
P38, P39	rs879255191	*LDLR*	19:11128090	c.2389+5G>A	-	ND	Conflicting interpretations of pathogenic/likely pathogenic
P50	NM_000527.4:c.(67+1_68-1)_(1586+1_1587-1)del	*LDLR*	-	-	-	ND	Eliminated a region spanning exons 2 to 10
P57, P58, P81, P103	rs121908038	*LDLR*	19:11113293	c.1202T>A	p.Leu401His	ND	Likely pathogenic
P65	rs137853964	*LDLR*	19:11129602	c.2479G>A	p.Val827Ile	A = 0.001006	Likely pathogenic
P70	rs570942190	*LDLR*	19:11113337	c.1246C>T	p.Arg416Trp	T = 0.000024	Not reported in ClinVar
P78	rs875989907	*LDLR*	19:11106666	c.796G>A	p.Asp266Asn	A = 0.000012	Pathogenic
	rs879254769	*LDLR*	19:11110765	c.1054T>C	p.Cys352Ser	ND	Likely pathogenic
P90	rs755757866	*LDLR*	19:11110730	c.1019G>T	p.Cys340Tyr	T = 0.000008	Likely pathogenic
P76	rs2147257524	*LDLR*	19:11116909	c.1756T>C	p.Ser586Pro	ND	Likely pathogenic
P23	rs773328511	*LDLR*	19:11106680	c.810C>A	p.Cys270Ter	T = 0.000008	Pathogenic
P25, P94	rs5742904	*APOB*	2:21006288	c.10580G>A	p.Arg3527Gln	T = 0.000275	Pathogenic
P96	rs145164937	*ABCG5*	2:43832056	c.293C>G	p.Ala98Gly	C = 0.002223	Conflicting interpretations of pathogenicity/likely pathogenic

## Data Availability

Raw data are available upon request from the corresponding author. These data are not publicly available due to privacy concerns.

## Abbreviations

CHD	coronary heart disease
CVD	cardiovascular disease
DLCN	Dutch Lipid Clinic Network
FH	familial hypercholesterolemia
HDL-C	high-density lipoprotein cholesterol
heFH	heterozygous familial hypercholesterolemia
hoFH	homozygous familial hypercholesterolemia form
LDL-C	low-density lipoprotein cholesterol
Me	median
MLPA	multiplex ligation-dependent probe amplification
TC	total cholesterol
TG	triglyceride
